# Community implementation of a brief parent mediated intervention for toddlers with probable or confirmed autism spectrum disorder: feasibility, acceptability, and drivers of success (IE Drmic et al.)

**DOI:** 10.3389/fped.2023.1295294

**Published:** 2024-01-23

**Authors:** Irene Drmic, Jessica Brian, Caroline Roncadin, Chantelle Shaver, Marlene Pase, Natalie Rugajs, Kristina Tofano, Erin Dowds, Lonnie Zwaigenbaum, Isabel M. Smith, Susan E. Bryson

**Affiliations:** ^1^ McMaster Children’s Hospital, Autism Program, Hamilton, ON, Canada; ^2^ Autism Research Centre, Bloorview Research Institute, Toronto, ON, Canada; ^3^ Department of Paediatrics, University of Toronto, Toronto, ON, Canada; ^4^ Autism Research Centre, University of Alberta, Edmonton, AB, Canada; ^5^ Department of Pediatrics and Department of Psychology & Neuroscience, Autism Research Centre, IWK Health Centre, Dalhousie University, Halifax, NS, Canada

**Keywords:** community implementation, implementation effectiveness, Exploration Preparation Implementation Sustainment (EPIS) framework, naturalistic developmental behavioral intervention (NDBI), Social ABCs, autism, community-partnered participatory partnership

## Abstract

**Background:**

Social ABCs is a caregiver-mediated Naturalistic Developmental Behavioral Intervention for toddlers with confirmed/suspected Autism Spectrum Disorder (ASD), with evidence in controlled research settings. Information is lacking on implementation in community settings. We reported on the treatment effectiveness of this program within a community setting, and the current paper describes the implementation phase of this work. Distinguishing between treatment and implementation effectiveness is critical for transporting interventions from laboratory to community.

**Objectives:**

Describe the implementation of Social ABCs through a large public autism service, supported by a research-community partnership.

**Methods:**

We describe this project through the Exploration, Preparation, Implementation, Sustainment (EPIS) framework as it focuses on implementation of evidence-based practices in publicly funded services. We apply this framework to the reporting stage. This project took place in the context of a 3-year government-funded pilot at a hospital-based publicly funded autism service. *Participants:* Program developers; Autism Service team; toddlers with suspected/confirmed ASD aged 14–34 months (*M* = 25.18 months) and their caregivers. *Training/supervision:* Provided by program developers at tapering intensity. *Evaluation:* Caregivers completed the Caregiver Diary and satisfaction surveys. We explored training processes, intervention uptake, acceptability, adaptations to fit community context, appropriateness, perceived impact, and facilitators/barriers.

**Results:**

Six coaches were trained to fidelity, and three of these were further trained as Site Trainers. 183 clinically referred families enrolled and 89.4% completed the 12-week program. Caregivers reported increases in adherence and competence, high satisfaction and perceived benefits for their children. Coaches reported high satisfaction. Toddlers were appropriately identified to receive the intervention. Referral processes improved, including decreased referral age, and increased family readiness for diagnostic assessment and subsequent services.

**Conclusions:**

Social ABCs was successfully implemented in a community service through a research-community partnership. The program was feasible, acceptable, and appropriate within a community context. Drivers of success included funding, institutional support, shared decision-making, adaptations to fit context, leadership support, perceived positive impact, and commitment to evaluation.

## Introduction

The past decade has evidenced an increase in research on the efficacy of interventions for toddlers with autism spectrum disorder [ASD; (1, 2)]. One prominent approach involves the application of behavior analytic teaching principles in naturalistic environments within a developmental framework [i.e., “naturalistic developmental behavioral interventions,” NDBIs; (3)]. Most NDBIs promote the involvement of primary caregivers, usually parents, to foster children's learning in the context of foundational relationships. However, the nature and extent of parent involvement varies across NDBI models; some are primarily therapist delivered with added parent involvement, while others are delivered exclusively by the parents or other primary caregivers (hereafter “parent-mediated”). Such approaches are not only developmentally well-suited for the toddler years, but they may also be particularly resource-efficient and thus appealing in resource-constrained systems (e.g., in contexts with limited funding for intervention services and reduced workforce capacity), and may be an ideal way to support families early (i.e., before a diagnosis is confirmed) in the context of long wait times for diagnosis and more intensive supports (4, 5). Recent meta-analytic findings concluded that “NDBIs have emerged as the intervention type most supported by evidence from RCTs” (1). However, a substantial research-to-practice gap remains, with persistent barriers to moving evidence-based interventions into community practice (6–10), including professionals’ self-reported limited knowledge and confidence in the efficacy of NDBI's (11).

The Social ABCs is a parent-mediated NDBI supported by evidence of *efficacy* from a tightly controlled randomized controlled trial [RCT; (12)]. In the standard, 12-week version of the program, all parent learning and practice takes place with a coach in the family's home or surrounding community setting (e.g., local playground). Parents’ learning sessions involve individual didactic instruction, supported by a Parent Manual, and practice-based learning that involves direct 1:1 (coach:parent + child) in-vivo coaching while the parent interacts with their child; parents are encouraged to integrate the strategies into their everyday interactions with their child, during play and family routines (note that no specific instructions are given about how much time to spend practicing between sessions, as the goal is for parents to use the strategies when they make sense and feel natural, within the family's multiple responsibilities and priorities). The main treatment targets are shared positive affect (i.e., shared smiles and mutual enjoyment between child and caregiver) and directed, intentional vocal communication. The *treatment effectiveness* of the Social ABCs has been demonstrated recently through a community implementation partnership (13). The current paper focuses specifically on the *implementation effectiveness* of that community partnership. Making a distinction between treatment effectiveness and implementation effectiveness has been identified as a critical step in transporting interventions from the laboratory to community settings (14).

The current paper describes the implementation of the Social ABCs through a large public regional autism service, supported by a research-community partnership (6, 7). We describe this initiative through the Exploration, Preparation, Implementation, Sustainment (EPIS) framework (15, 16), which focuses on the implementation of evidence-based practices in publicly funded services. Moreover, the EPIS framework has recently been used to examine ASD services specifically (7, 17). The EPIS framework has been applied in various ways in research studies, including exclusively in the analysis and/or reporting stage (16), which is our approach here. Although the framework is used here to guide discussion of the program roll-out across all phases, the main focus is on the implementation phase of this initiative.

## Methods

**Figure 1:**
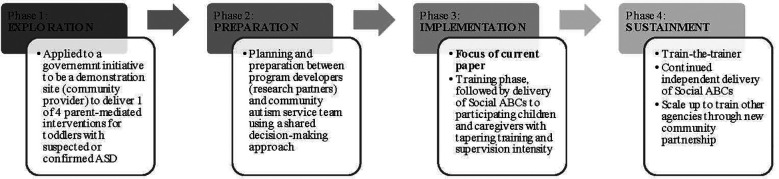
Applying the EPIS framework to provide an overview of the four phases of the social ABCs implementation process [adapted from (15)].

The EPIS framework was applied to the reporting phase of the Social ABCs community implementation. We use the framework to report on implementation outcomes, as well as facilitators and barriers relating to outer and inner context, and innovation/bridging factors. By way of context, Figure 1 depicts the four phases of the EPIS framework. Although each phase is briefly described, the focus of this paper is on the implementation phase.

### Phase 1. Exploration

The community delivery of the Social ABCs took place within the context of a publicly funded hospital-based clinical autism service. In this context, the Social ABCs program was funded by the provincial government of Ontario, Canada, as part of a “demonstration” program designed to evaluate the feasibility of community delivery of parent-mediated intervention models for toddlers with suspected or confirmed ASD. This government initiative was motivated by emerging evidence at the time that very early intervention, particularly parent-mediated approaches, can have a significant impact on toddler development, and may be a feasible way to support families while awaiting a diagnostic assessment. The seminal paper describing the concept and rationale for NDBI approaches (3) had just been published and there was growing motivation from community partners (service agencies, clinical and research experts, families) to support NDBI models for toddlers. In Ontario at that time, government-funded ASD intervention programs primarily involved traditional applied behaviour analytic (ABA) models, at relatively high intensity (referred to as “intensive behavioural intervention”; or “IBI” in the Ontario context). However, due to system constraints (insufficient financial and human resources to meet the growing need), long waiting lists had emerged and it was estimated in a 2013 report from the province's auditor general that most children with ASD would not receive ABA services before age six (18). Representatives of the provincial government had recognized a need for early intervention services for toddlers with emerging signs of ASD (ideally even before a diagnosis was confirmed).

In their 2017–2018 annual report, the provincial government (via the Ministry of Children and Youth Services; MCYS) announced that “The ministry is partnering with clinical experts and children's services organizations to demonstrate four new pre-diagnosis early intervention models in Ontario over the next three years. The pilots are play-based and are delivered in natural settings” (https://www.ontario.ca/page/published-plans-and-annual-reports-2017-2018-ministry-children-and-youth). This initiative was spurred by advocating families and the advice of clinical and research experts and service providers, via the ASD clinical expert committee that was mandated to “provide the ministry with expert advice on up-to-date and evidence-based research to help inform policy and program development”; https://www.ontario.ca/page/published-plans-and-annual-reports-2015-2016-ministry-children-and-youth. The four selected models (Early Start Denver Model, Early Social Interaction/SCERTS, JASPER, and the Social ABCs) were all NDBI approaches with some demonstrated evidence of efficacy, that were felt to be well-suited for implementation in Ontario's community-based services. Interested community agencies were invited to submit a detailed proposal, and members of the MCYS provincial committee selected four agencies to be part of the “demonstration.” Each agency was matched with a particular model, with a mandate to support staff training and deliver the program over the following three years (2016–2019; with the preparation and training for the current partnership having started late in 2016).

### Phase 2. Preparation

Once the [BLINDED] autism service was matched with the Social ABCs, the community-partnered participatory partnership began (19). An implementation plan, co-developed by the clinical service team and Social ABCs program developers, was submitted to the government sponsor. It outlined the planned referral process, eligibility criteria, intake and assessment plan, service delivery targets, waitlist management, parent involvement, integration with and transition to other services, staffing model, staff training and development, plan for communication and raising awareness, evaluation, work plan, and budget.

The clinical autism service team worked together with Social ABCs program development team to co-design minor *a priori* adaptations to the program to increase fit within the clinical service (vs. the previous research context). Adaptations included: (1) the clinical service would accept a wider range of toddlers than in previous research contexts [e.g., those in full-time daycare, and with co-occurring developmental challenges, which had been exclusion criteria in the previous research evaluation; (12)], and (2) caseload expectations were increased to meet clinical service targets.

The preparation phase also involved joint community awareness activities, starting with a program launch event to introduce the Social ABCs to the hospital community (families, clinicians, researchers, management), followed by outreach presentations to local service providers (e.g., preschool speech-language specialists, pediatricians). Information was posted on the hospital website and supported by media releases (i.e., local, national news). Finally, preparation for program evaluation included submissions to Research Ethics Boards at the clinical service and program developers’ institutions, to allow for formal outcome evaluation.

### Phase 3. Implementation

The implementation phase of the EPIS framework is the focus of the current paper. Here, we discuss the setting and participants, training and implementation methods, and implementation outcomes.

#### Setting

The intervention was delivered by the publicly funded autism service at [BLINDED] Children's Hospital, an academic health science center, in [BLINDED city] between November 2016 and October 2019. [BLINDED city] is a culturally, linguistically, and economically diverse city (population >500,000), with approximately 25% of residents born outside Canada (2016 Census/Wiki).

#### Participants

##### Autism service (clinical) team

*Parent Coaches* (hereafter “coaches”, 6 females, all Caucasian) were trained to coach parents or other primary caregivers (hereafter “parents”). Coaches were full time employees of the hospital-based autism service, previously behavioural clinicians (“Instructor Therapists”) in the IBI program. Educational backgrounds included Early Childhood Education (*n* = 2), Child and Youth Worker/Studies (*n* = 3), and undergraduate degrees in Psychology (*n* = 1). Three had additional college-level certificates in Autism and Behavioral Science. Years of ASD experience ranged from 7.5 to 15, with three coaches having 10 or more years. *Program Coordinator* (1 full-time equivalent; FTE) was a permanent employee of the autism service with 15 years’ previous experience as a behavioral clinician. The Program Coordinator organized all aspects of program management, including clinical duties such as intake, screening, identification of family needs, and referral management, organizational duties such as scheduling appointments, and data management and reporting. *Psychometrist* (0.5 FTE position) conducted psychometric assessments supervised by a psychologist. *Program Psychologist* (0.4 FTE position) provided clinical supervision, program evaluation, and supervised/ conducted assessments. *Leadership* representation at various levels (i.e., Program Director, Clinical Director, Manager, Clinical Leaders) provided operational oversight, reports to government, and advocacy for continued government support.

##### Program developers (research partners)

The *Program Co-Developer* worked with the Leadership team to plan the implementation and engaged in ongoing consultation with the clinical team, provided oversight for training, and led the research evaluation of program outcomes [see (13)]. *Social ABCs Psychologist* took on the role of clinical supervisor for the Lead Trainer. *Lead Trainer* provided on-site training and supervision of coaches (detailed below). Two additional *Trainers* provided initial training and participated in group supervision and video review.

##### Toddlers and caregivers

Toddlers (aged 12–36 months) with suspected or confirmed ASD and their caregivers (mostly parents) were referred to the intervention program by internal hospital clinicians, staff from external community services (i.e., physicians, speech-language pathologists, occupational therapists, infant-parent specialists), or were self-referred by parents seeking services. Eligibility criteria required that the family lived within the service catchment area and toddlers had an ASD diagnosis or related social communication concerns identified by clinicians or family and confirmed by interview, home visit, Infant-Toddler Checklist (20), and/or clinical judgement. The coached parent needed sufficient English proficiency to access the parent manual content (4th grade reading level) and follow live coaching in English.

Toddlers were ineligible if they had severe vision, hearing, or motor deficits (and were redirected to a more appropriate clinical service). No restrictions were placed on birthweight, gestational age, other neurological, genetic, or mild sensory or motor delays/conditions. Start of service was delayed until toddlers could hold up their heads and reach for objects, but no upper or lower limits were imposed on toddlers’ language development. Attendance at daycare was not restricted, but enrollment in other social communication or speech-language therapy programs was deferred during the 12-week Social ABCs coaching phase to minimize overlapping or incompatible treatment.

Characteristics of participating families are presented here briefly [for more details, see (13)]. Data were available for 179 (of 183) participating toddlers, yielding a sample of 72.6% boys, 12.3% born prematurely, 88.8% mothers (as coached parent) and 20.7% of parents describing themselves as English language learners. Mean age of referral was 22.9 months (range: 11–33 months), and age at program entry was *M* = 25.2 months (range: 14–34 months). At the beginning of the program, toddlers’ Receptive and Expressive language age equivalents (*M* = 10.14; range <1–30 months, and *M* = 12.53; range 4–26 months, respectively) were measured using the Mullen Scales of Early Learning (21). Overall adaptive function was captured using the Vineland Adaptive Behavior Scales–II, Adaptive Behavior Composite (22); *M* = 74.

#### Training and supervision of coaches

Training involved in-person didactic teaching (via initial workshop and supervision meetings in the hospital) and in-home “meta-coaching” (a technique developed by the Social ABCs program development team that includes in-the-moment instructions, cueing, reinforcement and encouragement to coaches while they coach families), as well as video review and discussion (in-person and through video-conferencing with the Lead Trainer and program development team) with tapering intensity. Fading of external supports occurred in tandem with increasing internal oversight by the hospital-based community clinical team (including peer-to-peer support), to build in program sustainability once the program development team concluded their involvement. The Lead Trainer was on site regularly (tapering from 4 days/week to 2 days/ month by the end of the demonstration partnership). Training and supervision were tailored for individual coaches, with additional supervision as needed or requested, and live feedback and/or video review of coaching fidelity.

All training and supervision intentionally mirrored the positive approach to coaching that is used with families in the Social ABCs program, consistent with the Pivotal Response Treatment [PRT; (23)] training model. Specifically, during coach training, strategies were introduced, discussed, and a rationale was provided, then coaches were supported (e.g., with scaffolding via in-the-moment “meta-coaching”) to ensure successful coaching opportunities, which were then reinforced with positive and specific feedback. The objective was to ensure coaches “got it right” from the beginning so they could immediately experience the impact of their coaching and receive positive feedback. If coaching errors were made, they were not met with corrective feedback—rather, the trainer would keep the error in mind and provide additional support on the next opportunity, in order to ensure success. The program developers embed this approach into all training and supervision activities, as it parallels the way that coaches will work with families, with an emphasis on parental empowerment, collaborative idea-generation, optimizing successful interactions, and supportive feedback. This collaborative and positive approach is foundational in Social ABCs parent coaching, informed by the recognition that many parents experience significant stress during the years surrounding their child's diagnosis (24), and that parenting stress can both interfere with learn­ing (25) and be mitigated through use of a collaborative approach [as reviewed in (26)].

Each of the following training steps is discussed below: Initial workshop, active training, program delivery (with continued supervision), and train-the-trainer.

##### Initial workshop

**Figure 2:**
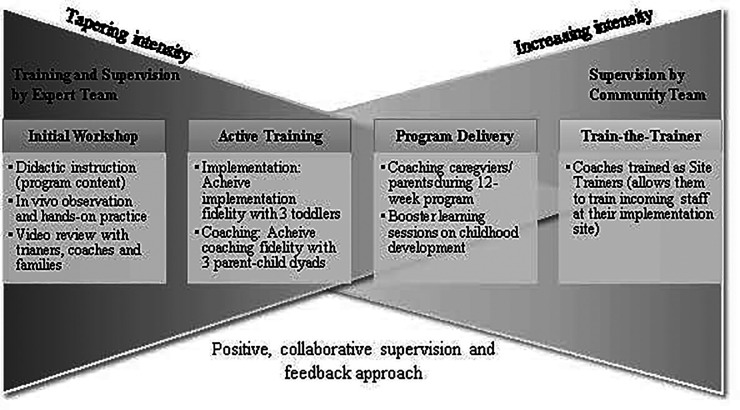
Training model.

Training began with a 5-day intensive workshop (25 h) led by the program development team (Co-developer JB, Psychologist AS, Lead Trainer ED, and two Research Trainers (SMW and KB). Each Coach-in-training was paired with a family (consented as “training families”) for the initial 12 weeks of training. The workshop entailed didactic teaching supported by video examples, demonstration of how to “talk families through” the manual, and direct practice implementing the intervention with families (with *in vivo* support via live coaching and video review). Additional content-related learning opportunities (e.g., booster didactic sessions on toddler development, understanding tantrums) took place at annual in-person meetings. Training stages are described in Figure 2.

##### Active training

Coach training involved two practical components: (1) direct program delivery with the child (implementation), and (2) training in parent coaching. Note that direct implementation of the Social ABCs by coaches is used only as an initial training strategy and is not a program component once a coach is fully trained. Gaining experience in direct implementation is felt to help coaches-in-training understand and experience the coaching techniques that they will eventually use with parents and allows them to gain perspective of how it feels to be coached in an active, positive, moment-by-moment manner. Direct implementation entailed hands-on practice in the home with the toddlers, with active support from the trainer. The training target entailed achieving fidelity of implementation (i.e., >80% accurate delivery of program components directly with toddlers) across three families. While practicing directly with toddlers, trainees also began to practice coaching caregivers, again with moment-by-moment guidance (“meta-coaching”) from trainers.

Following sufficient practice in coaching (described in results), new trainees were evaluated for their fidelity of coaching with caregivers. Coaching fidelity was evaluated by the program development team, using a modified version of the PRT train-the-trainer fidelity form (23). Modifications were co-designed with the program development and clinical program team to meet their learning needs. Our target for coaching fidelity was >80% correct use, across three families, of five specific coaching elements [i.e., providing specific feedback, clear and concise direction/feedback, focus on positives/successes, moving from suggestive-to-directive feedback as required, and focus on priority issues; adapted from PRT; (23)].

In addition to each trainee's work with their assigned families, the first three months of training also entailed trainees attending each other's coaching sessions to gain more exposure to a range of child and parent learning styles, peers’ coaching styles and techniques, and to hone their skills in observing and supporting their peers. This peer-to-peer support was carried throughout the partnership within the context of joint supervision meetings and video review once it was no longer possible (due to increasing caseloads) to attend each other's coaching sessions.

##### Program delivery

During program delivery, Coaches delivered the Social ABCs to families through the clinical service, with tapering support and oversight from the program development team. Throughout the partnership, coaches worked with a total of 183 families, 179 of whom provided outcome data for the evaluation of treatment efficacy [reported in (13)]. The intervention entailed 12 weeks of in-person, in-home Social ABCs coaching [as described in (12, 13)], delivered by one of six coaches. Parents were coached to use strategies that enhance toddlers’ functional communication and shared positive affect, and to integrate strategies into daily caregiving routines and playful interactions. Following three months of tapered coaching, caregivers were encouraged to keep using the strategies without additional input from coaches and were invited to return for follow-up after an additional three months.

##### Train-the-Trainer

Four coaches were identified to receive additional training to become Site Trainers so that they could train new staff following the end of the partnership [based on a Train-the-Trainer model that promotes program sustainability; e.g., see (27)]. Three coaches completed this phase (one took parental leave and was not able to complete this level of training). This training involved achieving fidelity of implementation and coaching and demonstrating proficiency in training at least two new coaches, with minimal support from the program development team. The Lead Trainer observed and provided feedback to the emerging Site Trainer on the manual overview delivered to families and meta-coaching of the new staff. Once coaching fidelity was achieved, it was not reassessed throughout the program; however, regular quality monitoring occurred during supervision sessions with the Lead Trainer. Moreover, per published work, parent fidelity was used as a proxy measure of the quality of the coaching (28).

#### Data collection and analysis

##### Program adaptations

First, we describe program adaptations that were made before and during the implementation.

##### Demographics

Demographic data were collected at intake, including date and age at referral, diagnostic status at entry, gestational status, daycare attendance, and whether parents identified as English language learners; this information is reported elsewhere (13).

##### Feasibility metrics

As an index of *program feasibility*, information was collected from the clinical service, such as number of toddlers referred, deemed eligible, agreeing to participate, as well as those who started, completed (including number of weeks completed), and dropped out of the program (reason documented). This information is reported in more detail in Brian, Drmic et al. (13). *Feasibility of training* was examined by tracking training hours for each coach-in-training, including activities such as the Lead Trainer observing and supporting while trainees provided module review and live coaching with families (i.e., “meta-coaching”), supervision meetings, and time spent video-coding.

##### Appropriateness

To explore whether the toddlers who participated in this service were appropriately identified for the intervention, diagnostic information regarding autism and other diagnoses was collected.

##### Acceptability and perceived impact

To explore factors of acceptability, we collected formal feedback from caregivers and invited informal reflections from coaches, caregivers, and other service providers from the hospital.

*Caregiver acceptability* was measured using the *Social ABCs Satisfaction Survey* (12). Caregivers completed this 6-item questionnaire post-intervention, rating items from 1 (strongly disagree) to 5 (strongly agree). Parents were asked to rate their satisfaction with the program overall, the live coaching, their coach's responsiveness to questions/concerns, and the manual, as well as perceived child gains. Mean scores are an index of overall satisfaction with the program. Following the specific questions, families were invited to write their qualitative reflections in an open-ended section. An overview of these reflections is presented in the results, but this input was not subjected to formal thematic analysis.

*Perceived Impact* was measured using the *Caregiver Diary* (29). This measure was used to examine caregiver-rated perceptions of the intervention (i.e., “buy-in”) via program adherence and competence, and whether caregivers or others noticed any developmental progress in the child. The Caregiver Diary asks the parent to report their experience with the strategies being taught. Four questions address caregiver adherence (e.g., “an issue for me is… finding the time to carry out the strategies; …that the strategies are complex/ difficult/ do not feel natural; …that I have to put in a lot of work to carry out the strategies”) and two address caregiver competence (“I am still not very confident/ comfortable with the strategies”). Each item is rated from 1 (not at all) to 5 (very true), with higher scores indicating more difficulty. One question asks about change in the child (“have you noticed your child interacting differently?”), with 1 indicating “no difference at all” and 5 indicating “definite differences”. In year one, data were collected weekly from weeks 2 to 8 (excluding training families). In response to families’ reports that weekly collection interfered with therapeutic time, collection was reduced to weeks 2, 4, 8, and 12 thereafter.

*Coaches* Feedback from *Coaches* was collected informally through written reflections about their experience solicited at project end. As above, this feedback was not subjected to formal thematic analysis, but is presented as a sampling of reactions from these sources.

*Internal Service Providers* (i.e., developmental pediatricians, non-Social ABCs autism clinicians) were also invited to share written feedback; these reflections are presented below but were not subjected to formal thematic analysis.

## Results

**Figure 3:**
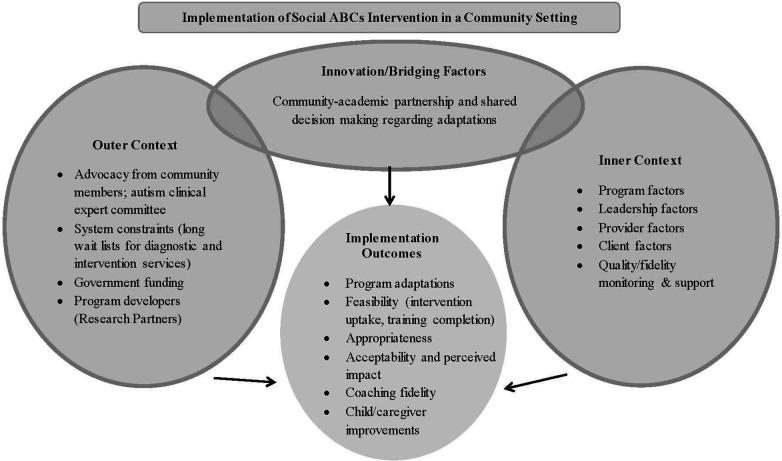
Infuence of outer context, inner context, bridging and innovation factors on outcomes of the EPIS framework [adapted from (17)] in the context of community implementation of the social ABCs.

The EPIS framework is used to highlight implementation outcomes and key contextual factors (barriers and facilitators). See Figure 3 for application of the EPIS framework to the Social ABCs implementation project.

### Implementation outcomes

#### Program adaptations

As outlined above, deliberate, *a priori* adaptations were made during the Preparation phase of the project, including (1) broadening inclusion criteria, and (2) increased case load, both to align with the needs of the community government-funded service delivery context. Two additional adaptations were made during the training phase and program delivery. First, to support training activities, the teams co-designed a Coaching Fidelity form, adapted from the Train-the-Trainer fidelity form developed for PRT (23). The final adaptation involved an adjustment to the coaching schedule to better accommodate families’ and coaches’ schedules. Specifically, families’ work/daycare schedules were accommodated by offering early morning and evening home appointments, and a more flexible treatment schedule compared to the previous RCT (i.e., adjusting from the original model of 3, 2, and 1 visits in the first three weeks, respectively, to a 2, 2, 2 schedule). Coaches reported that families, particularly working families, found this schedule more manageable; moreover, this somewhat more regular schedule was felt to be easier for caseload management and planning for staff scheduling.

Most adaptations (all but the coaching fidelity form) were initiated by the service delivery team, highlighting the need for inclusivity, staff scheduling consistency, and a relatively high caseload within a government-funded clinical program. The modifications were supported by the program developers, recognizing the need for accountability and given that the proposed modifications were sufficiently minor as to not threaten the integrity of the program (i.e., up-front intensity of learning and a tapered coaching schedule was felt to be essential for parent learning, but the modified schedule retained that approach).

Widening the eligibility criteria yielded enrollment of a diverse population, including 5 sets of twins (all 10 received the intervention), toddlers born preterm (<36 weeks gestation; 12%), parents/caregivers who worked full- or part-time (*n* = 65 full-time; *n* = 8 part-time), and for whom English was not a first language (i.e., English Language Learners; 20%), children attending daycare (28%) or with grandparents as primary carers (4%), and with more clinical complexity (e.g., dual diagnoses).

#### Feasibility

##### Intervention uptake

Of 253 toddlers referred for the Social ABCs intervention program, 183 were enrolled for service. Of the 70 who were not enrolled, 35 did not meet eligibility criteria (i.e., lived outside of the clinical service catchment area, lacked social communication signs on screening, or required an English interpreter), 19 declined, and 16 could not be contacted. Of the 183 eligible, we had access to data from 179 families. Of those, 160 (89.4%) completed the 12-week program, 9 families completed eight weeks of the program, 6 completed four weeks, and 4 withdrew early in the program (i.e., after one week or less). The average number of families that did not finish ranged from 5.6% to 12.5% (*M* = 8.6%) across fiscal quarters.

##### Training

All 5 coaches-in-training achieved fidelity of implementation and coaching above 90%; an additional (sixth) coach was trained by Trained-Trainers during the Trained-Trainer learning phase. Mean training hours for each coach to achieve fidelity was 96 h, taking place across a six-month period (mean duration = 119 days). The sixth coach received 154.5 h of training (longer duration to accommodate Site Trainers’ learning needs).

Program outcomes (e.g., parent fidelity, toddler responsivity, performance on clinical assessment measures) are beyond the scope of the current paper, and are described in detail elsewhere (3).

#### Appropriateness

The process of referral and intake resulted in enrollment of toddlers who were appropriate for the Social ABCs program. Of the 179 families willing to share clinical data, diagnostic information was available for 175 toddlers. As noted above, the vast majority of these toddlers did not have a confirmed diagnosis when they were enrolled into the program (i.e., only 11% came in with a confirmed ASD diagnosis), but the majority did end up with an ASD diagnosis (an additional 50% received a diagnosis during or shortly after program completion). Diagnoses were made by qualified professionals at McMaster Children's Hospital or in surrounding community practices. As described in Brian, Drmic et al. (13), just over 30% of participating toddlers had no confirmed diagnosis at the conclusion of data collection but were still being investigated for ASD. Taken together, it can be estimated that up to 90% of participating toddlers may have ASD (11% received a diagnosis before enrollment, 50% during or shortly after participation, and an additional 30% were still awaiting diagnostic confirmation). Small numbers had other confirmed/suspected diagnoses (language delay, Down syndrome, global developmental delay, genetic finding, and query ADHD). Only two toddlers enrolled in the program were discharged from service with no diagnosis despite having had social communication challenges at intake.

#### Acceptability and perceived impact

##### Program level impact

Referrals initially came through central hospital intake and were directed into one of three pediatric clinical programs, and then into the Social ABCs. Later, referral processes were refined and referrals came directly into the Social ABCs program (as appropriate) with co-referral to other programs to maximize efficiency of service coordination. For example, speech-language services were postponed while families received Social ABCs; once complete, families were redirected back. As the program matured, the Program Coordinator also facilitated referrals to other services, such as daycare, occupational and physical therapy, developmental assessments, and technology access clinic. As the referral process become more streamlined, the average age of referral decreased from 30.8 months (year 1) to 22.1 months by year 2, and 20.1 months by year 3. In addition to the more direct referral processes, another major contributor was a slight change in eligibility criteria during the project to accept children only up to 30 months at start of intervention (vs. 36 months in the first year). To accomplish this, the program coordinator worked with intake and referral sources to ensure that younger children were referred in a timely manner and would not “age out” of service.

##### Caregivers

Caregivers reported high levels of satisfaction with the program (*M* = 4.9/5; *n* = 80); including the live coaching component (*M* = 5.0/5), their coach's responsiveness (*M* = 4.9/5) and perceived gains in child language (*M* = 4.5/5) and child smiling (*M* = 4.6/5). Caregivers reported that the parent manual was helpful (*M* = 4.6/5), and they also found it to be helpful for the coach to talk through the manual with them (*M* = 4.9/5). Caregivers reported liking various aspects of the intervention, including that it was caregiver-mediated and the techniques were found to be helpful, easy to learn, and felt natural. One caregiver reported, “*I love this program so much. It's so natural…she's learned so much and I’ve learned so much.*” Caregivers reported increased confidence and competence using and incorporating the strategies into daily life. One parent shared, “*The coach helps build confidence gradually so that at the end of the 12 weeks you feel good about continuing on your own.*”

Based on scores on the *Caregiver Diary* (*n* = 100)*,* parents reported improvements, from week 2 to week 12, for program adherence (*M* = 2.40, *SD* = 1.00 vs. *M* = 1.60, *SD* = .79) and competence (*M* = 1.90, *SD* = .88 vs. *M* = 1.17, *SD* = .46), and identified toddler developmental progress (*M* = 3.33, *SD* = 1.12 vs. *M* = 4.31, *SD* = .67). Paired samples *t* tests comparing week 2 vs. week 12 were all significant: *t* = 8.68, 8.47, and—0.13 for adherence, competence, and child change, respectively, all *p*'s < .001).

A collateral benefit of earlier access to the program (prior to obtaining a diagnostic assessment) was that coaches often supported families through the diagnostic assessment journey (recall that almost 50% of families received an ASD diagnosis while participating in the program). Within this context, coaches were able, with parental consent, to provide diagnostic clinicians with detailed information based on direct observations of the child and their response to treatment. They could also help prepare parents for what to expect from the assessment process, and in some cases families asked them to attend assessment appointments with them, which may be a testament to the trust that had been fostered through the coaching relationship. One element of the training includes helping parents interpret and understand their child's unique strengths and challenges, often within the context of (probable/emerging) ASD. The notion of “autism literacy” (a term coined by Lead Trainer, E Dowds) emerged as an important concept, wherein Social ABCs families were felt to be increasingly ready for clinicians’ questions, reflections, and feedback during the diagnostic assessment process, in response to having participated in the program (see quote from Developmental Paediatrician, below, regarding families being “primed” for the diagnostic assessment process). See Appendix for more examples.

##### Coaches

Based on informal feedback from coaches, they all expressed being satisfied with the program *(*“*It's amazing to know that I have provided a parent invaluable training at such an early stage*”). They also described the positive impacts on caregivers, including increased skills and confidence in interacting with their children, positive outlook on their children's futures, improvements in bond with their children, and that parents were empowered by the children's successes. One coach stated that “*Social ABCs gave parents a glimpse of what their child is capable of, and what they as parents are capable of as well, and I think this is very powerful.*” Another coach reflected on families’ increased readiness for diagnosis, sharing: “*I think it was beneficial to families to have support from a clinician at this time point, preparing them for the appointment and/or reflecting with them after it occurred. The majority of families I served had never heard of the word Autism before Social ABCs. From my experience parents developed a better understanding of ASD and how their child learns.*” See Appendix for more examples.

##### Other service providers

Developmental pediatricians also provided informal feedback, reporting perceived increases in caregiver competence and empowerment: “*As a diagnostic clinician, I found that the families of children who had participated in the Social ABCs come with a clearer understanding of the purpose of a developmental paediatrics consultation (in most cases) as they have been* “*primed*” *with the right language and its understanding as it pertains to describing/identifying areas of social communication”.* Clinicians from the behavioral autism service reported that the families who participated in Social ABCs came into ABA therapy with a good understanding of ASD and the ABC (antecedent-behavior-consequence) model of learning and behavior. These clinicians felt that, following the Social ABCs, caregivers started behavioral services with an increased expectation, readiness, and confidence to participate in their children's ABA program. One autism interventionist shared, “*I feel that parents who have gone through the Social ABCs program have a clear understanding that their participation in ABA services is essential to their child's growth. The families have an expectation that they are a member of the treatment team. These families are experienced and are wonderful to work with.*” See Appendix for more examples.

### Contextual factors

Contextual factors related to putting Social ABCs into practice in a community setting are outlined below, with consideration of whether they were facilitators or barriers. These factors were considered informally in the context of training and implementation of the program.

#### Outer context

An early key facilitating factor was the competitive application process, which ensured institutional buy-in and investment in the program from the beginning. Additionally, funding from the government supported staff during the training phase (an up-front investment to promote program quality), and the government mandated an appraisal of feasibility (i.e., numbers of children referred, enrolled, completed, etc). Other facilitators included the commitment of the program development team to increasing access to the program by building community capacity, and their proximal location (approx. 85 kilometers from the clinical program site). Conversely, the instability of long-term funding presented a barrier at the conclusion of the demonstration phase, resulting in loss of some trained staff and negatively impacting sustainment.

#### Inner context

*Program-level factors* such as a positive learning climateand internal program champions played important facilitating roles. The clinical team championed the programcolleagues. The dedicated full-time Program Coordinator, with clinical ASD experience, was a facilitator in terms of supporting clinical (e.g., intake, screening, referrals, and supporting families) and administrative (i.e., scheduling appointments, data collection, reporting) functions. Moreover, this individual also participated in the early training phase. We do not have the evidence to claim that this was a kay variable in this person's proficiency with service navigation, but her deep understanding of the program was likely a facilitator in triage and referral processes.

*Autism service leadership* played a facilitating role in the implementation. This included a formally appointed internal implementation leader (program psychologist) and an opinion leader (Clinical Director), both with high buy-in, and clinical as well as research experience. The opinion leader generated interest and excitement across the clinical service and in the broader community, identified gaps and needs, shared research evidence with other members in leadership positions, and advocated for parent-mediated intervention to front-line clinicians.

Specific *provider factors* that facilitated program success were staff “buy-in” and perceived “fit” (of the staff and of the program itself). This was fostered by the invited recruitment of front-line staff who were highly motivated to learn the new intervention (buy-in), and their skill sets/ professional perspectives were felt to be a good fit by the management team for a caregiver-mediated program. Staff buy-in has been identified as a key factor influencing the success or failure of innovation uptake [see (30)]. While the coaches’ extensive experience working with children with ASD was a facilitator, a barrier that required mitigation (via additional training modules) was the relative inexperience of working with toddlers and limited knowledge about early development A barrier early in training, was an initial reluctance of trainees to be video-recorded and observed by the training teams. However, trainees quickly reported feeling comfortable due to the positive and supportive supervision model and relationship with the Lead Trainer, together with an emerging recognition of the value of the video review as a training tool. Staff turnover related to parental leave was both a barrier and facilitator, in that new staff were recruited and trained. This involved increased time and resources, but this also provided the opportunity for coaches to gain experience as Site Trainers (i.e., by training those new staff members), thus facilitating future sustainment of the program.

Various *client factors* were seen as facilitators, including fit of the intervention with the developmental needs of toddlers [cf (30).], and the caregivers’ positive engagement and high satisfaction with the program. Another key facilitator reported by caregivers was that the intervention was easy to incorporate into daily routine activities. One major barrier was that the program was only provided in English.

*Quality/fidelity monitoring and support* involved built-in ongoing fidelity checks, and coaches were trained to conduct their own and peers’ fidelity checks as the program development team faded their support. The positive training model facilitated coaches’ comfort with peer mentorship, setting the tone for the intervention model and the expected interactions with caregivers. Coaches initially reported finding it difficult not to receive (or provide) corrective feedback, as they had previously worked within a Behavior Skills Training framework in which corrective feedback is applied [i.e., identification of “areas that need improvement”; (31)]. Over time, however, coaches stopped requesting corrective feedback and described a positive shift in how they worked with families, provided feedback to colleagues, and even in their personal interactions. This positive coaching approach was seen as central to the success of the implementation.

#### Innovation factors

Social ABCs, like many interventions, was developed and initially evaluated under controlled research conditions with narrow inclusion criteria. Collaborative engagement with the program developers facilitated appropriate adaptations to improve fit within a community context, including broadening inclusion criteria andadaptations to the intervention schedule allowing for more flexible delivery. The lack of a coaching manual for coaches was identified as a barrier.

#### Bridging factors

The collaborative working relationship that involved shared decision-making between the program developers and clinical team throughout all phases of the partnership was an important facilitator allowing the program to be adapted to fit into the community service model. The program development team supported various aspects of evaluation, from development of the evaluation plan, submission to the Research Ethics Boards, and program evaluation. The program development team obtained external research funding to examine and report on treatment effectiveness (13).

### Phase 4. Sustainment phase

To facilitate sustainment, Social ABCs uses a trained-trainer model, wherein coaches can be trained to a level qualifying them to train new coaches at their organization. The trained-trainer model has been used in similar programs to support program spread across large regions and sustainment over time [e.g. (27),]. One key sustainment barrier was the external (government funding) context. Following the 3-year demonstration project, funding was not extended, which resulted in two trained coaches leaving the program. However, because the intervention was seen as valuable, and the investment in training had been substantial, community leaders advocated for renewed government support, eventually securing an additional year of funding. The COVID-19 pandemic then emerged, necessitating adaptation for virtual delivery—the team's ability to pivot to virtual program delivery involved minimal consultation with the program developers (i.e., two 90-minute sessions). Virtual program delivery is not described here (since the demonstration project had concluded by then) but pilot findings evaluating the virtual model (in an abbreviated, group-based learning format) show promise (28). At the time of writing, a new government initiative has allowed for further program sustainment and expansion that is currently underway.

## Discussion

The current paper describes an implementation collaboration between intervention program developers and a large community service agency, using a community-partnered participatory framework. Findings illustrate the feasibility and impact of using hybrid effectiveness/ implementation designs to promote the adoption of evidence-based practices into clinical care (7, 32). We used the EPIS framework to report on the process and outcomes, with a focus on the Implementation phase of the EPIS cycle, and identified barriers and facilitators to success.

Implementation outcomes demonstrated that ASD interventionists from an intensive behavioural intervention service could be successfully trained as Social ABC coaches (reaching fidelity targets) in approximately six months, with a subset trained as Trained-Trainers (now called Site Trainers) to support program sustainment. Although coaches came with significant experience in ASD and use of behavioral intervention techniques, knowledge gaps in early child development were identified and supported with additional learning modules. This reveals that staff who lack a theoretical background in early development may still become skilled coaches in the toddler sphere, as long as enhanced training is provided to fill this knowledge gap. At the time of the demonstration project, many frontline ASD therapists in Ontario had had limited experience with toddlers due to long wait times for diagnosis and entry to service. However, with recent advances in access to early parent-mediated models for toddlers, the hope is that this will continue to improve over time; the addition of developmental theory in training courses for autism intervention specialists may also be a way to enhance knowledge in this area. Coaches reported a positive training experience, and liked the gradual, tailored and non-corrective (positive) coaching and supervision model and supportive and safe relationship that was developed with the Lead Trainer. This positive model of coaching was an important factor that set the tone for the intervention throughout the partnership and was felt to create a positive shift in culture. Coaching staff described this shift as being like “Shangri-La”, and positively impacting interactions with caregivers and colleagues, as well as in personal life interactions (personal communication).

The training model was extensive (3-year collaboration) and intensive (involvement of program development team and on-site Lead Trainer, both at tapering intensity), which fostered a solid understanding of the training elements and processes necessary for successful delivery of the program in a community context. Although successful, the current training model has a high resource burden, and may need refinement to increase efficiencies (i.e., to reduce costs) and to increase reach to more remote or hard-to-access communities. This collaboration provided the opportunity to identify key training facilitators and barriers that have already begun to inform subsequent training initiatives. Several innovations have emerged in response to feedback from coaches in the current training partnership, including the development of a coaching manual, and the establishment of a community of practice. An abbreviated coach training schedule has now been developed that maximizes efficiency by leveraging the group-based Social ABCs model (28) to allow for practice with a higher volume of families over a shorter duration. Evaluation of this training model is currently underway.

The program was feasible for delivery within a large community autism service. Feasibility refers to the extent to which an innovation can be successfully carried out within a novel setting (33), based on factors such as recruitment, participation, and retention rates. The intervention was delivered to 183 eligible families (almost 90% of whom completed the 12-week program), the number and breadth of referral sources increased over time, and streamlined referral processes led to efficient, integrated, and supportive care pathways. One of the most impactful process changes was that the age of referral decreased over time (by almost 10 months), thereby optimizing access for the children for whom the program was intended (i.e., those under 3 years of age). The screening process yielded appropriate referrals; namely, infants/toddlers with confirmed or probable ASD, most of whom did eventually receive an ASD diagnosis of or were being monitored for ASD at the conclusion of the program.

An unanticipated benefit for families was their increased readiness for the ASD diagnosis. This is related to a concept we describe as “ASD literacy” which includes parents’ understanding their child's strengths and challenges, increased hopefulness related to developmental gains made during therapy, a closer parent-child bond, and supportive relationships with coaches throughout the program. Literature has shown that during the diagnostic process parents may feel confused, uncertain, and uninformed about ASD and appropriate services and treatments (34, 35), and that receiving the diagnosis can be an intense emotional experience (35–37). Indeed, the lead up to a diagnostic assessment (i.e., “the undercurrent of anticipating”) has been characterized as a particularly stressful stage in the diagnostic journey (37). Thus, receiving the diagnosis from a position of preparation, empowerment and support may mitigate at least some less favourable aspects of the experience. A remaining question is whether this sets parents and children on more optimal trajectories. Indeed, family-centered care models have been linked with increased parent satisfaction, decreased parent stress, and improved child outcomes (38). Based on a recent scoping review of parents’ experiences of the ASD diagnosis, satisfaction has been positively correlated with factors such as the professional's reactions to parents’ first concerns (39), information provided at diagnosis (39–41), and post-diagnostic supports (40). Families involved in the Social ABCs were supported by coaches who heard and validated parental concerns and supported the areas of need, identified strengths, observed developmental gains, and facilitated access to needed services and next steps.

Caregivers reported acceptability of the program via high satisfaction ratings, increased confidence and competence, and they reported developmental progress in their children. Parental buy-in and self-efficacy are essential for parent-mediated interventions to be effective (42, 43). Coaches and caregivers both described developing a positive caregiver-coach partnership. Coaches appreciated being part of families’ journeys, from navigating day-to-day issues to facilitating paths to care. A collaborative family-centered relationship has been linked with less stress and higher levels of parenting competence (44).

### Impact of contextual factors on implementation

Collaboration between researchers and community partners is central to moving interventions effectively from research into community-based care (45, 46). A key driver of the successful partnership was the collaborative working relationship that involved shared decision-making throughout all phases of implementation. For instance, decisions about adaptations were made jointly by the community and research teams. Adaptations to improve fit of an intervention program to the setting increase the likelihood of intervention adoption and sustainability (8, 45, 47), and can be “intentional” or “unintentional” (48). Intentional adaptations were made in partnership during the Preparation phase to increase program fit and reach. An unplanned adaptation to the schedule was made in response to feedback from caregivers to improve fit and maintain engagement, while retaining dosage. Adaptations and ongoing problem solving occurred through formal (i.e., meetings, yearly review) and informal (i.e., impromptu in-person, phone, or email commination) avenues for discussion.

Government sociopolitical and funding contexts influence consideration and uptake of innovation (7). The impetus for adoption of the program was a government initiative to explore the feasibility of expanding services to toddlers with confirmed or suspected ASD, given a growing recognition of the need for earlier, developmentally informed and caregiver-mediated interventions. This supported early buy-in from organizational leadership and set the expectation and provided funding for training and partial evaluation. Empirical evidence supports the importance of engagement of leadership at all levels for effective implementation (49–51).

Two key sources of leadership influence supported the program. The first came from an opinion leader (Clinical Director) who influenced the attitudes and beliefs of upper management and front-line staff, and supported communication among all levels of staff. Although evidence supporting the effectiveness of opinion leaders as health-care change agents is limited and results are mixed (52, 53), we considered this an implementation facilitator. Second, a first-level leader (internal clinical team Psychologist) facilitated implementation and communication between community and research teams. Evidence suggests that first-level leaders are important in health services and in a position to influence intervention implementation (54, 55). Furthermore, provider buy-in and attitudes towards innovation affect use and sustainment (56, 57).

In this partnership, buy-in from coaches was also high from the start related to readiness for a new learning opportunity, and was bolstered by the positive work environment and high levels of satisfaction. Coaches also felt well-equipped to provide the service and reported that the program was a good fit for the developmental stage of the toddlers with whom they were working and their families. Staff “buy-in” and perceived “fit” of a program have been identified as facilitators of uptake of evidence-based educational practices in ASD (30).

However, staff stress and dissatisfaction were also noted, related to uncertainty regarding job security (i.e., continued government funding), and two coaches left for other job opportunities once the government funding became less secure. Sustained funding after initial implementation is a key factor in public service sectors (6).

### Strengths and limitations

The Social ABCs caregiver-mediated program for toddlers was successfully implemented by a large hospital-based autism service, and implementation outcomes were collected, including acceptability and feasibility metrics, informal feedback from staff and community clinicians, and identification of facilitators and barriers. Limitations of this work included the informal collection and presentation of feedback from staff and partners (i.e., we did not conduct formal qualitative analyses and our quantitative measures were limited to caregiver feedback and did not include coaches and service providers). Although this feedback provided important insights, informal data collection and measurement introduced the potential for positive bias. Ideally, recorded content of focus groups or meetings would have been analyzed by independent coders, but our evaluation plan did not allow for that level of analysis. An additional limitation was our lack of formal implementation measures to examine contextual factors (e.g., provider factors, organizational climate). However, processes were carefully monitored throughout the three-year partnership, and stakeholder feedback was invited and documented, allowing us to explore the influence of contextual factors less formally. We applied the EPIS framework retrospectively to report on the process, outcomes, and influence of contextual factors, an approach that has also been employed by others [for review see (16)]; however, future application should use the framework prospectively at earlier planning phases when factors and processes are being assessed and to operationalize components (16). Future research would benefit from more rigorous implementation science research methodology and measurement.

### Conclusions and guidance for program planning

The current findings illustrate the feasibility and impact of using hybrid effectiveness/ implementation designs to promote the adoption of evidence-based practices into clinical care (7, 32). It has been demonstrated that an intervention aimed at early social communication concerns in at-risk infants and toddlers can be delivered in a community setting. Providing services to very young children with emerging signs of ASD is important in light of evidence that very early intervention has positive effects on development [e.g. (58),] and emerging evidence that early effects may lead to enhanced long-term outcomes (1, 59).

Key facilitators included joint decision-making regarding any program adaptations (to maximize feasibility whilst retaining program integrity), program champions, careful selection of staff with high buy-in and fit, a positive learning environment, ongoing (tapering) support and supervision, streamlined referral pathways and processes, and open and transparent communication between staff and program management teams. Common barriers to implementation of ASD interventions in the education sector include constraints associated with resources, time, consistency of program delivery (i.e., quality control, program fidelity), staffing factors such as low buy-in, lack of support from other program personnel and leadership, and lack of training (30). The current partnership successfully mitigated some of these common barriers by ensuring program quality and consistency (through rigorous training and fidelity measurement), protected time for learning, support from program leadership, and staff buy-in), with the main barrier emanating from external factors (specifically, unstable government funding).

Expansion of the Social ABCs is currently underway, fueled by a new government initiative to increase access to caregiver-mediated early years services across community agencies in Ontario (see https://www.ontario.ca/page/ontario-autism-program-caregiver-mediated-early-years-programs). This new initiative aims to scale up community access to the intervention, with funding awarded for capacity building (e.g., increased staff training, and, at least in the case of the Social ABCs, enhancing training of Trained-Trainers so they can build capacity within their local communities with increasing independence from the program development team), thus reducing costs and increasing capacity. Lessons from the implementation project described in this paper have informed revised processes (e.g., development of a coaching manual, refined evaluation of coaching fidelity) that will improve and formalize the training model and serve to identify contextual factors important to successful implementation, scale-up and sustainment.

## Data Availability

The raw data supporting the conclusions of this article will be made available by the authors, without undue reservation.

## Appendix 1 Informal Reflections from Caregivers, Coaches, and Other Service Providers

Caregiver Impacts

1) *High Satisfaction with the Social ABCs intervention* was reported by caregivers and coaches. Caregivers reported liking various aspects of the intervention, including that it was caregiver- mediated and techniques were helpful, easy to learn, and felt natural. One caregiver reported, “*I love this program so much. It's so natural…she's learned so much and I’ve learned so much.*” One Coach shared, “*It's amazing to know that I have provided a parent invaluable training at such an early stage.*”
2) *Caregiver empowerment.* Caregivers reported increased confidence and competence using and incorporating the strategies into daily life. One parent shared, “*The coach helps build confidence gradually so that at the end of the 12 weeks you feel good about continuing on your own.*” Coaches described the positive impacts on caregivers, including increased skills and confidence in interacting with their children, positive outlook on their children's futures, improvements in bond with their children, and that parents were empowered by the children's successes. One coach stated that “*Social ABCs gave parents a glimpse of what their child is capable of, and what they as parents are capable of as well, and I think this is very powerful.*”
3) *Support provided by coaches.* In general, a positive and supportive relationship was developed between the caregivers and coaches. Caregivers reported high satisfaction with their Coaches and noted that the support provided by the Coaches was invaluable. One parent said, “*Our trainer was a great motivator and pointed out many things that made so much sense and helped me understand my son's behaviours better*.” Coaches also valued being part of the journey with families and described building partnerships with caregivers.
4) *Increased parental readiness for diagnosis.* Coaches and developmental pediatricians reported that parents were better able to recognize areas of concern in their child, were more accepting of the possibility of a diagnosis, and had a good understanding about what to expect during the diagnostic assessment appointment. Coaches helped validate parental observations and helped prepare and support families for the diagnostic journey. One coach shared: “*I think it was beneficial to families to have support from a clinician at this time point, preparing them for the appointment and/or reflecting with them after it occurred. The majority of families I served had never heard of the word Autism before Social ABCs. From my experience parents developed a better understanding of ASD and how their child learns.*” Developmental pediatricians also reported increased caregiver competence and empowerment: “*As a diagnostic clinician, I found that the families of children who had participated in the Social ABCs come with a clearer understanding of the purpose of a developmental pediatrics consultation (in most cases) as they have been ‘primed’ with the right language and its understanding as it pertains to describing/identifying areas of social communication*.
5) *Increased parental understanding and involvement in subsequent behavioral intervention services.* Clinicians from the behavioral autism service reported that the families who participated in Social ABCs came into ABA therapy with a good understanding of ASD and the ABC (antecedent-behavior-consequence) model of learning and behavior. Caregivers started behavioral services with an expectation, readiness, and confidence to participate in their children's ABA program. One autism interventionist shared, “*I feel that parents who have gone through the Social ABCs program have a clear understanding that their participation in ABA services is essential to their child's growth. The families have an expectation that they are a member of the treatment team. These families are experienced and are wonderful to work with.*”

Perceived Child-level Impact

6) *Child-level developmental gains.* Improvements in children's skills were noted by caregivers, Coaches, and other clinicians. One Developmental Pediatrician said, “*There were obvious improvements in communication from time of referral to date of consultation. It was to the point that I stopped reading the consultation request information until after my initial assessment, because children had made such impressive gains in such a short time*.” Caregivers shared that their own understanding and bond with their children increased, with one stating, “*I understand her more and our bond keeps growing. Without Social ABCs, it may have taken years to build that bond. I truly believe Social ABCs kick-started her learning and the amazing bond we now have*.”

Impact on Coaches

7) *Positive training experience*. Coaches liked the positive, flexible, tailored, and gradual (from observation to implementation to coaching) coaching and supervision model. One Coach said, “*Being part of the Social ABCs has been a great learning experience and was like a breath of fresh air.*” Coaches described the benefits of implementing Social ABCs with toddlers before coaching parents because it put them in the parent's shoes and allowed them to understand how hard it is to motivate children. Another coach shared, “*I believe that implementing before coaching is essential in training a successful parent coach.*”
8) *Increased job satisfaction*. All Coaches, who previously worked in traditional ABA-based services for many years, reported increased job satisfaction and a positive working environment. One Coach reflected, “*This has been the most rewarding job I’ve ever had,*” and Coaches often described the experience like “being in Shangri-La.” Coaches described learning new skills (e.g., early signs, using a developmental lens, providing feedback in a positive manner, how to discuss concerns) and increased professional confidence (e.g., making clinical decisions). One Coach shared, “*I learned so much in such a short period of time. This program really rekindled my love for supporting families and children.*” At the outset, a large pool of applicants applied to be Coaches, particularly because it afforded the then-unique opportunity to learn a parent-mediated NDBI model. Job instability for coaches was a concern throughout as the government funding was time-limited; despite this uncertainty, Coaches remained committed and positively engaged in the program.
